# Influence of Binaural Beats Stimulation of Gamma Frequency over Memory Performance and EEG Spectral Density

**DOI:** 10.3390/healthcare11060801

**Published:** 2023-03-09

**Authors:** Ludymila Ribeiro Borges, Ana Paula Bittar Britto Arantes, Eduardo Lazaro Martins Naves

**Affiliations:** 1 Assistive Technology Laboratory (NTA), Faculty of Electrical Engineering, Federal University of Uberlândia, Uberlândia 38400-902, Brazil; 2 Hotchkiss Brain Institute (HBI), Cumming School of Medicine, University of Calgary, Calgary, AB T2N 1N4, Canada

**Keywords:** binaural beats, power spectral density, working memory

## Abstract

Similar to short-term memory, working memory cannot hold information for a long period of time. Studies have shown that binaural beats (BB) can stimulate the brain through sound, affecting working memory function. Although the literature is not conclusive regarding the effects of BB stimulation (stim) on memory, some studies have shown that gamma-BB stim (40 Hz) can increase attentional focusing and improve visual working memory. To better understand the relationship between BB stim and memory, we collected electroencephalographic data (EEG) from 30 subjects in 3 phases—a baseline, with gamma-BB stim, and control stim—in a rest state, with eyes closed, and while performing memory tasks. Both EEG data and memory task performance were analyzed. The results showed no significant changes in the memory task performance or the EEG data when comparing experimental and control conditions. We concluded that brain entrainment was not achieved with our parameters of gamma-BB stimulation when analyzing EEG power spectral density (PSD) and memory task performance. Hence, we suggest that other aspects of EEG data, such as connectivity and correlations with task performance, should also be analyzed for future studies.

## 1. Introduction

Memory functions are associated with oscillations of electrical activity in the brain and can be classified into long and short-term. Working memory (WM) is not completely distinct from short-term memory and refers to the temporary storage and manipulation of small amounts of information over brief periods of time [1]. Literature suggests that cortical theta, alpha, and gamma bands play a mechanistic role in multiple aspects of memory. This role includes the representation and offline maintenance of events and sequences of events, the assessment of novelty, the induction of plasticity during encoding, and the consolidation and retrieval of stored memories [2].

Neural synchronization in the gamma frequency is often linked to short-range communication within brain areas, while long-range communication relates to neuronal phase locking in slower frequency bands [3,4]. In line with this characteristic, increased gamma-band power improved the top-down control of feature bindings [5].

As a noninvasive brain stimulation method, binaural beats (BB) are characterized as a slight difference of frequencies played simultaneously to the ears, stimulating a brain wave in a frequency proportional to the difference of frequency. For example, if 400 Hz plays in the right ear and 440 Hz on the left, the brain will be entrained in 40 Hz. Oster [6] applied this concept to medical conditions in 1973.

BBs are referred to as central beats because the interaction of the auditory stimuli most likely occurs in the superior olivary nuclei in the brainstem [7]. Neurons in the brainstem are sensitive to phase shifts between both ears. When these phase shifts occur, the brainstem neurons fire action potentials that correspond in rate to the phase difference between both ears [8].

Wang et al. [9] showed that listening to 15 Hz binaural beats is a proven intervention for mental fatigue that can contribute to maintaining working memory function. Moreover, participants who underwent a 12-min BB stimulation of 9.55 Hz significantly increased their working memory capacity [10]. Another controlled study showed that listening to 15 Hz, BB increased response accuracy on a visual-spatial working memory task [11]. However, in one study, healthy participants listened to a 7 Hz (theta) BB for 30 min, and the immediate recall memory was significantly decreased in the experimental condition compared to the control condition [12].

A variety of cognitive functions, among them memory, involve gamma-band activity. Moreover, gamma frequency is related to the binding process within sensory processing, attentional enhancement of sensory input, and both working and long-term memory [13]. The frequency of gamma in synaptic transmission is related to inhibitory (GABAergic) and excitatory (glutamatergic) activity [14]. The mechanism for the selective transmission of sensory information across different brain areas seems to be based on gamma frequency [15]. Gamma also works as a neuroprotective, influences nonneuronal cell types, and affects synaptic plasticity, learning, and memory [16]. In patients with Alzheimer’s disease (AD), interareal gamma coherence and gamma power are involved [17]. The same occurred with mice models of AD, which presented reduced abnormal gamma activity [18].

Regarding Mujib et al. [19], Jirakittayakorn & Wongsawat [20], Wahbeh et al. [12], Gao et al. [21], and Corona-Gonzalez [22], the current literature presents contradictory findings. While some report that BB has shown to be successful in practice [19,20], other BB studies have achieved no electroencephalographic (EEG) modulation (brainwave entrainment) [12,21,22]. Table 1 summarizes the methodology approach of some studies that analyze BB and memory found in the literature.

Although specific studies support BB as a promising method for cognitive enhancement, major issues, such as incompatible methodological approaches, remain to be addressed. The protocol of the stimuli application has not been well established in the literature. The effect of brief BB stimulation on scalp EEG has not been conclusively demonstrated, especially in gamma frequency. Given the technique’s growing popularity among neuroscientists, the current study adds further substance to the growing literature approaching memory modulation with BB.

We hypothesize that the noninvasive stimulation with BB in gamma frequency masked with classical music can change brain activity and improve working memory performance. The novelty aspect of our study is the analysis of working memory performance and the EEG signal with new approaches for signal processing under gamma-BB frequency stimulation. Providing detailed documentation about the method, we aim to obtain significant results that can serve as parameters for other studies, as well as the development of new rehabilitation techniques.

## 2. Materials and Methods

### 2.1. Experimental Design

We recruited 30 healthy adults (18 to 33 years old, 9 males) for a placebo-controlled, cross-over, double-blind trial. All volunteers were assessed using the Montreal Cognitive Assessment test (MOCA) as inclusion criteria (M = 25, SD = 2). The volunteers had no neurological or psychiatric history, such as epilepsy, seizures, attention deficit hyperactivity disorder, or hearing or vision deficiency. They were not taking any medication at the time of the experiment. They also abstained from alcohol and caffeinated beverages 12 h before the testing sessions.

The audio files were created using the software Audacity (version 1.2.3). For the experimental stim condition, two sine tones of 400 and 440 Hz were played separately and simultaneously in the right and left ear, respectively, to produce 40 Hz BB. The BBs were masked with the classical music track La Stravaganza by Antonio Vivaldi. The control stim condition consisted of the same classical music without the BB. The audio files were saved in uncompressed *.wav* format to avoid frequency distortion during the encoding process. An uninvolved person named the recordings to support a double-blind experiment. The auditory stimuli were played through a stereo headset (Sony EH-1000XM3) with noise canceling.

The volunteers sat facing a computer while we collected their EEG signals throughout the experiment. The experiment was divided into three phases: without stim, with the experimental stim, and with the control stim. In each phase, we collected EEG data both in a rest state, with eyes closed, and while performing memory tasks. The experiment took approximately 2 h, including the setup process.

The first phase, without any auditory stim, began with the EEG baseline collection in a resting state, with eyes closed, for 2 min. Subsequently, the participants performed two memory assessment tasks in alternated sequences to avoid any order effect. The two tasks were

- Digit span task: A commonly used measure of short-term memory [1]. First, a random sequence of numbers appears on the screen. Second, users press the numbers on the keyboard in the same order the numbers were presented. The first tentative starts with one digit (0–9), incrementing by one digit after a correct answer in each trial. The performance is indicated by the highest number of digits correctly remembered. The test ends after two consecutive errors.
- N-back task: Continuous recognition measures present visual stimuli in sequence. The volunteer judges whether the current target matches the one that appeared N item back for each item. This protocol has 3 phases: 1, 2, and 3-back. Each phase consists of 60 trials. We saved the scores for the target (when they should press a keyboard button, confirming the target matches) and non-target (when they should not press a button because the target does not match) across the three phases and total.

**Figure 1:**
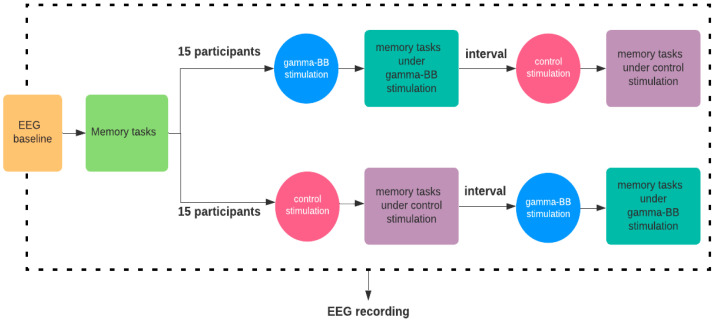
Diagram of the experimental protocol.

In the stim phase (second and third phases), the volunteers were equally and randomly allocated to a group. The first group started with gamma-BB stimuli. They listened to the prepared audio recording for 10 min with their eyes closed, then performed the memory tasks (Digit Span and N-back). After a 5-min interval, the subjects were presented to the control stim for 10 min, with eyes closed, and then performed the same memory tasks. The second group had this sequence inverted. The auditory stimuli continued until the tasks were completed in each phase. The order of the two memory tasks was presented on a rotating basis known as “Latin Squares” to prevent any carryover effects from one task to another. Figure 1 illustrates the experimental procedure.

The amplifier device used to record the EEG signals was the EEGO™ rt, with a waveguard™ original cap of 64 electrodes in 10/20 configuration from ANT Neuro corporation. We connected the ANT-Neuro device through the OpenViBE platform to acquire the signal at a sampling rate of 1024 Hz. A notch filter of 60 Hz was applied to mitigate external interference. Then, the EEG signal was saved in two extensions: *.ov* (OpenViBE extension) and *.csv* (Comma-Separated Values extension) for posterior analysis in Matlab. All electrode-skin impedances were maintained below 10 kΩ during the experiments.

### 2.2. Pre-Processing Stage

We applied an interactive template matching and suppression (ITMS) procedure in the pre-processing stage to detect and suppress blink artifacts [28]. The ITMS approach estimated a waveform of the blink-artifact after ten interactions from the original blink-artifact template resampled at the sampling rate of the EEG device (1024 Hz). Then, the blink-artifact model was suppressed from the raw EEG data. The ITMS algorithm is single-channel interaction-based. We applied the algorithm to the electrodes from the frontal lobe [29], channels: FP1, FPz, FP2, AF7, AF3, AF4, and AF8. Then, the data were filtered using a designed FIR filter, order 400, with cut-off frequencies of 0.1 to 100 Hz. Additionally, we proceeded with a visual inspection to confirm that no movement artifacts remained.

### 2.3. Features Extraction

We extract the power spectral density (PSD) using the sine-multitaper approach described by Barbour and Parker [30]. This approach is a recursive method suited to long time series, in which parameter tuning such as time bandwidth or segment length is not required. We extracted the relative PSD for the delta (0.1 to 4 Hz), theta (4 to 8 Hz), alpha (8 to 12 Hz), beta (12 to 30 Hz), and gamma (30 to 80 Hz) range.

In order to compare the experimental to the control stim, without and during tasks, the features—PSD and task scores—of the phase without stimulation were subtracted from the stimulation phase (experimental and control). The learning effects that can be caused by the performance of memory tasks were repeatedly decreased by Latin squares rotation and the standardization by the phase without stimulus.

We performed the Principal Component Analysis (PCA) to reduce dimensionality using the algorithm of Singular Value Decomposition (SVD), the default mode of the Matlab function *pca()*. This function extracts data in the directions with the highest variances. We have several matrices, 30 × 64 (one matrix for each condition and each frequency band), composed by the PSD values, where 30 was the number of volunteers, and 64 was the number of channels. We reduced to matrices 30 × 1 (the first component representing all channels). Then, we stored the first component of each phase (10 min stimulation and memory tasks under gamma-BB stim and control stim). We also extracted five channels that most contributed to the first component.

### 2.4. Statistical Analysis

We performed the non-parametric permutation test (50,000 permutations) using the first component and PSD of the channel that most contributed to the first component considering all conditions. Then, we compared these features for gamma-BB stim and control stim in a rest state, with eyes closed, and while performing memory tasks; across the delta, theta, alpha, beta, and gamma frequencies range. All the groups had the same size (30 samples). We also compared the resting state phases without any working memory task, with eyes closed, to the resting state with gamma-BB stim and control stim.

Subsequently, we detected the outliers using a boxplot with outliers labeled in Jasp software version 0.16.4.0 in the first component and the PSD of the channel that most contributed to the first component. We compared the groups again after excluding the outliers from both groups in order to keep the same size.

Finally, we also compared the memory task scores under experimental to control stimuli using the non-parametric paired test Wilcoxon, correcting for multiple comparisons and controlling for false discovery rate (FDR). We used the Matlab function *fdr_bh()*.

## 3. Results

**Figure 2:**
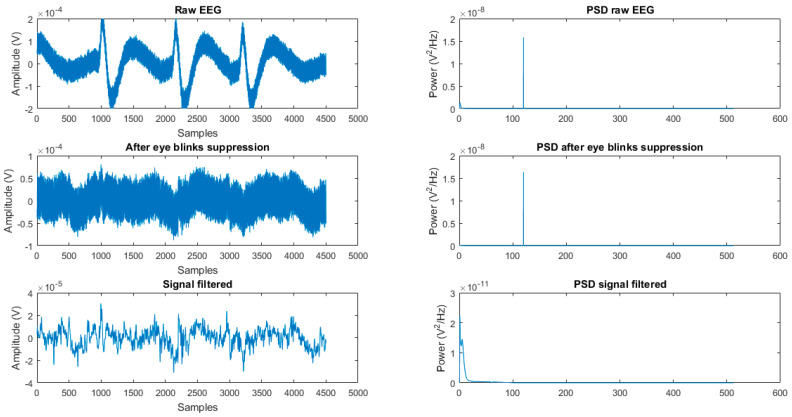
Results of the pre-processing stage in the signal and its respective PSD.

Figure 2 shows the raw EEG signal and the results after applying the ITMS algorithm and filtering in the pre-processing stage.

**Figure 3:**
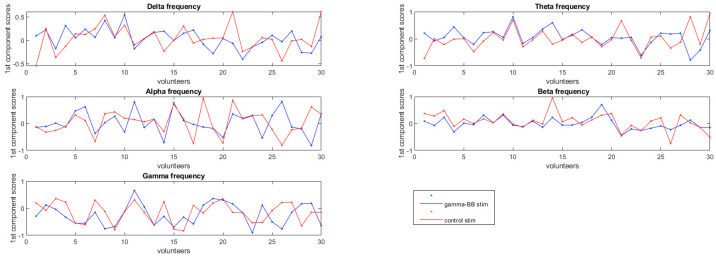
Plot of the scores of the first component per volunteer for the stimulation phase in a rest state across the bands (delta, theta, alpha, beta, and gamma).

Figure 3 shows the first component scores per volunteer in each frequency band. Table 2, Table 3 and Table 4 show the five channels that most contributed to the first component, how much this component explains the data, and the *p*-values for the permutation test using the first component. Channel F2 was the most highlighted across all conditions.

**Figure 4:**
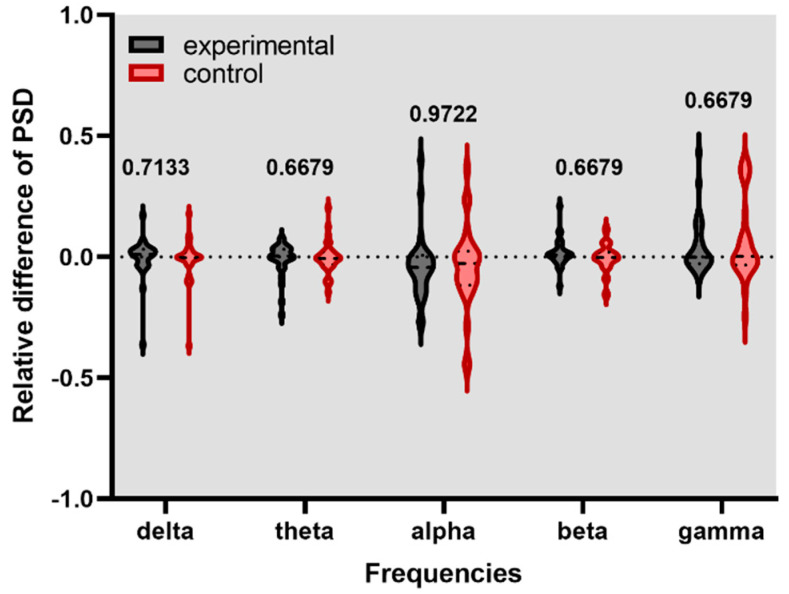
Relative difference of PSD for F2 channel during experimental and control stimulation without a task in a rest state.

**Figure 5:**
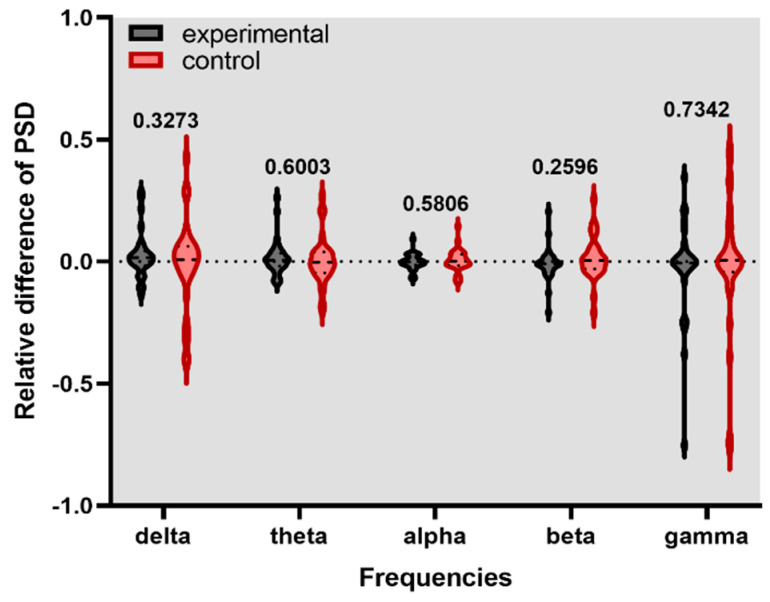
Relative difference of PSD for F2 channel during the digit span task.

**Figure 6:**
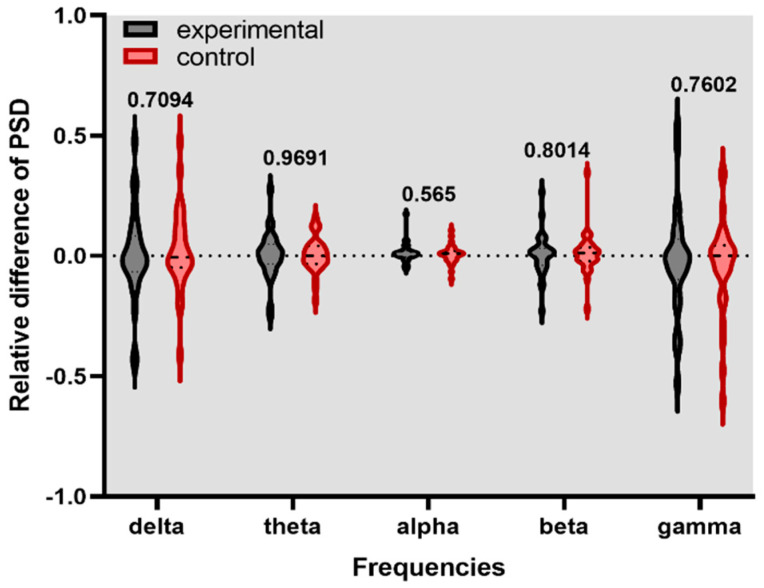
Relative difference of PSD for F2 channel during the N-back task.

Figure 4, Figure 5 and Figure 6 show the violin graph of the relative PSD of channel F2 in experimental and control conditions across the delta, theta, alpha, beta, and gamma frequencies and their respective *p*-value. The positive values of the y-axis mean higher energy for the stim phase compared to the without stim, while the negative values indicate the opposite.

**Figure 7:**
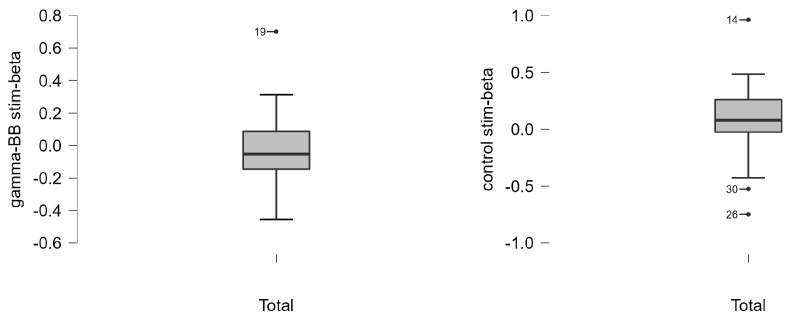
Boxplot of 1st component in resting state in beta frequency with outliers labeled for gamma-BB and control stim.

The groups did not present differences considering the first component and channel F2, nor after outlier detection and exclusion for any condition. Figure 7 exemplifies how we identified the outliers using a boxplot in Jasp software.

**Figure 8:**
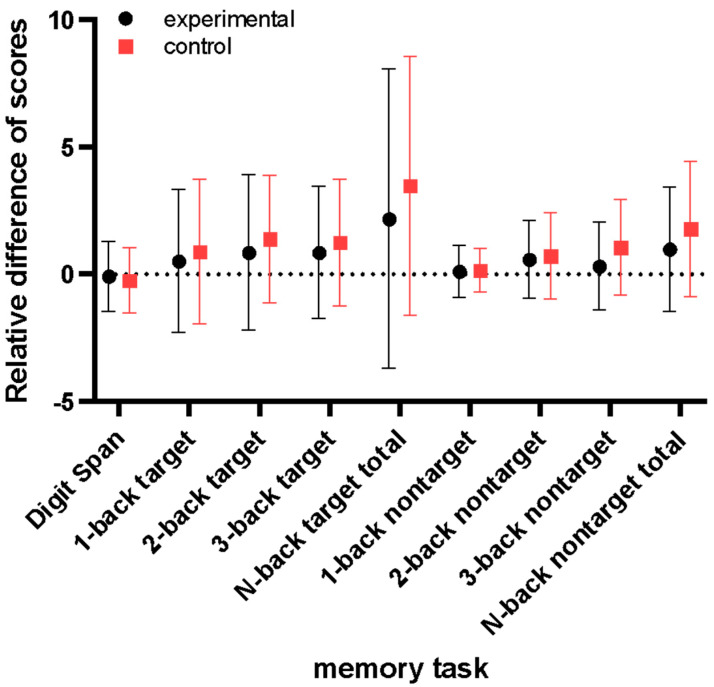
Relative difference of task score for experimental and control stimulation condition.

Figure 8 shows the results for the memory task scores. The positive values of the y-axis mean a higher score for the stim phase compared to the without stim, while negative values indicate the opposite. The results were not significant after correction for multiple comparisons and FDR.

## 4. Discussion

BB is a technique that is growing in popularity among neuroscientists. Among the sparse and controversial studies found in the literature, only Mujib et al. [19] investigated gamma-BB stimulation with EEG and working memory performance analysis, similar to our approach. However, our study presents some improvements, such as the presence of a control condition with a potential placebo effect, besides a detailed cross-over experiment with new approaches for signal processing.

This study investigated if brain entrainment could be achieved by gamma-BB stimulation and if the working memory could be enhanced. The analyzed features showed that after listening to either experimental or control stimulation conditions, no significant changes occurred in the PSD of the F2 channel and the first principal component. It suggests that brain entrainment was not achieved in gamma-BB stimulation.

Interpreting the effects induced by BB stimulation is a complex task since the BB stimuli have been assessed using different variables (neurophysiological and cognitive-behavioral) with heterogeneous methodologies. It is still unclear whether BB stim leads to a frequency following the response of the presented frequencies or whether it evokes different responses in the brain. Some studies report brain entrainment with BB stim. Jirakittayakorn and Wongsawat [20] showed that the FFT absolute power of gamma oscillation increased with time, especially in the frontal and central regions of the brain after the participants were exposed to the 40 Hz BB. The greatest induced changes were found within 15 min of listening. Similarly, Mujib et al. [19] found significant differences in the group with BB stimulus at 30 Hz. The authors observed an increase in the frontal gamma band power during the stim stage and both theta and gamma power increase in the bilateral frontal and left parietal cortex post-stim state.

Additionally, Corona-Gonzalez et al. [22] found no significant differences between two BB stimulation (theta and beta BB) sessions in brain entrainment. Similarly, in Wahbeh [12], there were no significant differences between the experimental (BB frequency in 7 Hz) and control conditions in any of the EEG measures. Furthermore, Gao et al. [21] identified no apparent brainwave entrainment effect. However, connectivity changes were detected following relative power variation during BB stim. Their observation supports that functional brain connectivity under BB stim is worth further study.

Brain activity during BB stimulation mainly influences the signal phase since neurons sensitive to time delays are primarily located in the brainstem, where their activation power is weaker [7,25]. As Gao et al. [21] reported, these effects suggest that BB could affect functional brain connectivity, but not necessarily by inducing a frequency-following response.

To better understand the mechanism of BB stimulation and modification in brain networks following memory enhancement with BB stimulation, it is vital to study the brain regions involved in processing working memory and auditory stimulation [31]. Although we did not find significant results comparing the PSD of the F2 channel, this channel seems important for the memory process, especially in theta frequency [32]. This channel might be important for memory because the frontal cortex is believed to be a source of top-down control of cortical sensory activity during goal-directed behavior. The medial prefrontal cortex has extensive evidence of increased spectral power during mental efforts, such as heightened attention required for short-term memory encoding. Studies applied auditory stimuli in the gamma frequency in humans and produced gamma entrainment in the frontal area [33,34]. Also, topographical distributions in Scholz et al. [35] suggest the generation of memory-related low beta oscillations in medial and frontal neural structures.

Contrary to what we expected, we found no evidence of any influence of BB on working memory scores. Some studies, such as Khattak [23] and Sharpe et al. [24], found memory enhancement after gamma-BB exposure. The study of Khattak [23] considered a larger sample size (N = 60) with an equal number of female and male participants. The participants who listened to the gamma frequency performed better on the word-free recall test than on the white noise condition. Sharpe et al. [24] did an exploratory pilot study with nine participants. The participants performed eight sessions over four weeks of pre- and post-exposure evaluation to a 5-min long binaural beat. They considered 25, 40, and 100 Hz for BB stimulation, and the memory score improved at a greater significance for 40 Hz. Considering the methodology of this paper, as the PCA technique reduces dimensionality through a linear combination in the directions with the highest variance, it considers different channels and weights to compose the first component. Thus, when we compare the first components, we have differences in the spatial source dependence that can lead to no significant results.

Analogous to the present study’s findings, Shekar et al. [26] did not find a statistically significant improvement between listening to the BB frequency of alpha and gamma and a constant tone of 340 Hz on memory task scores. However, they found a significant decrease in auditory and visual reaction time after entrainment with alpha and gamma binaural beats. This study had an equal number of female and male participants (N = 40), thus controlling for possible sex-related differences in hearing binaural beats [6]. It suggests that BB can enhance attention, not specifically memory performance.

Engelbregt et al. [25] compared white noise (WN), 40 Hz gamma BB and 40 Hz gamma monaural beat (MB). The participants (N = 24) performed two tasks under WN, BB, and MB stimulation conditions in a within-subject cross-over design. The authors masked the MB and BB conditions with white noise. They did not find significant results for working memory measured by Klingberg task scores. However, they found reduced reaction times for attention measured by the Flanker task in BB and MB conditions.

Moreover, Jirakittayakorn [20] found that the average numbers of words recalled by each participant before and after listening to the stimulus were not significantly different. In their protocol, the word list recall task was conducted before and after listening to the gamma-BB (40 Hz) for 20 min. However, after BB stimulation, better scores were found in remembered words at number 8.

Mujib et al. [19] evaluated participants’ short-term memory performance through digit span tasks under alpha (10 Hz), beta (14 Hz), and gamma (30 Hz) BB stimulation. They found increases in the cognitive score for the alpha condition, while a significant decrease in reaction time was noted for alpha and gamma.

Even though we did not analyze the reaction time feature, the studies conducted by Engelbregt et al. [25], Shekar et al. [26], and Mujib et al. [19] support the notion that faster attention processing may be attributed to the influence of gamma-BB. However, these studies did not find evidence that gamma-BB significantly influences working memory. Therefore, they further substantiate our findings.

Most studies found in the literature did not report whether the BB stim was embedded with a masking stimulus. In contrast, others masked the stimulation with white noise, pink noise, piano music, or natural sounds [10,12,25,27,36]. Inspired by Lim et al. [27] and Kraus & Michaela [10], we masked the BB stim with music to amuse the long protocol; it is worth highlighting it is a common way to find BB stim available on the internet. However, unmasked BB stim expressed larger effects than those masked with music [37]. It can be one of the reasons for not achieving brain entrainment through our approach in PSD analysis.

## 5. Conclusions

As a noninvasive method, BB stim can be easily applied for rehabilitation purposes. However, the role of BB stimulation in the brain is still unclear, and the frequency choice for memory enhancement is not trivial. Our results did not present significant differences between experimental and control conditions for working memory performance and PSD features.

In this context, few studies examine the relationship between auditory binaural stimulation and memory in healthy people. They present different approaches, such as frequency choice, duration of stimulation, study design, and assessment tool for working memory. Despite some studies containing similar approaches, many aspects are still unclear. The importance of the time the effect of auditory beats may persist is still unknown. Furthermore, whether BB in the gamma band elicits brain entrainment or alters brain connectivity is unclear. Which BB frequency works best to enhance memory performance and which carrier tones are most appropriate is also an unclear aspect.

Finally, the current study added further substance to the growing literature. Specifically, it presented a detailed and reproducible method describing the effects of BB in gamma frequency for working memory task scores and PSD. We also combined different approaches for signal processing and data analysis. For further studies, we suggest triggering the tasks so it would be possible to analyze different phases of working memory, such as encoding and retrieval. In addition, connectivity analysis and correlations with task performance would also characterize working memory under gamma frequency stimulation.

## Figures and Tables

**Table 1:** Characteristics and intervention protocols of some studies that approached BB and memory found in the literature.

Study	BB Stim (Hz)	Sessions (Exposure Time)	BB before or during Task	Carrier Tone (Hz)	Masking	Control Condition	Assessment Tools/Methods
Mujib et al., 2021 [19]	10, 14, 30	1 session (15 min divided into 3 sub-sessions with 5 min)	before	400 and 410, 400 and 414, 400 and 430	N.R.	none	digit span task, EEG
Khattak, 2021 [23]	40 (N.R.)	1 session (5 min)	before and during	450 and 410	N.R.	white noise	word free recall test
Sharpe et al., 2020 [24]	25, 40, 100	8 sessions (5 min)	before	N.R.	N.R.	none	mathematical problems, recall tasks
Engelbregt & Deijen, 2019 [25]	40	1 session (N.R.)	during	440 and 480,	white noise	white noise	flanker task; Klingberg test
Shekar, et al., 2018 [26]	10 and 40	1 session for each condition (N.R.)	N.R.	N.R.	N.R.	constant tone (340 Hz)	auditory reaction time, visual reaction time, short-term memory test
Lim et al., 2018 [27]	10 and 7–4 (N.R.)	1 session (20 min) for each condition with a 1-week interval	Before	N.R.	piano music and natural sounds	mechanical massage and relax (without acoustic stimuli)	d2-test, digit span test, Corsi block-tapping test, picture recognition test, EEG
Kraus & Michaela, 2015 [10]	9.55	1 session (12 min)	before	230 and 220.45	Music with BB	music without BB	Automated Operation Span Task
Wahbeh, et al., 2007 [12]	7 (133 and 140)	1 session (30 min)	before	133 and 140	Pink noise resembled the sound of rain	rain sounds	Rey Auditory Verbal List (RAVLT), Profile of Mood States (POMS), State-Trait Anxiety Inventory (STAI), Controlled Oral Word Association Test (COWAT), blood pressure, spectral and coherence analysis on EEG

N.R. = not reported.

**Table 2:** The five channels contributed most to the PCA first component, total variance explained by the first component, and *p*-values for permutation test results for experimental and control stimulation in a rest state.

Freq.	Gamma-BB Stimulation without Task	Explained (%)	Control Stimulation Without Task	Explained (%)	*p*-Value
Delta	F2, F3, F1, Fz, F4	61	F2, Fz, F4, F6, F8	58	0.5560
Theta	FC6, FC5, C5, FT7, C6	84	F2, AF7, F7,FCz, Fz	72	0.6317
Alpha	TP8, P2, PO8, Pz, C1	79	P2, Pz, PO8, PO6, PO4	79	0.9798
Beta	Pz, P1, P2, C1, Cz	72	C5, C3, C1, PO7, P1	76	0.2670
Gamma	C5, C6, FC5, C3, FT7	79	C1, CP4, CP3, TP8, Pz	76	0.7234

**Table 3:** The five channels contributed most to the PCA first component, total variance explained by the first component and *p*-values for permutation test results for experimental and control stimulation during the digit span task.

Freq.	Digit Span with Gamma-BB Stimulation	Explained (%)	Digit Span with Control Stimulation	Explained (%)	*p*-Value
Delta	F7, FC5, F1, F3, FC3	52	F2, Fz, F1, F3, F7	70	0.1737
Theta	FC3, F1, FC1, F2, C3	61	AF3, AF8, F2, Fz, AF4	69	0.4415
Alpha	C5, C4, C3, CP3, FT7	56	PO6, PO8, P8, O1, P6	72	0.6782
Beta	AF4, AF3, FCz, F2, FC3	71	F2, FP1, Fz, F1, AF4	74	0.2295
Gamma	C3, FC3, F1, CP2, P2	64	Fz, F2, AF3, F1, FCz	68	0.6192

**Table 4:** The five channels contributed most to the PCA first component, total variance explained by the first component, and *p*-values for permutation test results for experimental and control stimulation during the n-back task.

Freq.	N-Back with Gamma-BB Stimulation	Explained (%)	N-Back with Control Stimulation	Explained (%)	*p*-Value
Delta	F2, F7, F8, FCz, F1	83	F2, FCz, F7, Fz, F1	75	0.8736
Theta	FPz, FP1, FCz, AF4, AF3	79	AF4, FCz, AF3, F2, FPz	63	0.9725
Alpha	CP3, P5, P3, CP5, FC6	67	P4, CP3, CP1, P2, CP4	55	0.3467
Beta	F7, F2, FCz, AF8, FC2	73	F2, Fz, FCz, F1, C2	79	0.8759
Gamma	FC2, FCz, F2, C2, Cz	80	FCz, C2, P2, F2, C1	71	0.9506

## Data Availability

The data presented in this study are available upon request to the corresponding author. The data are not publicly available due to ethical restrictions.
